# Enhanced Thermal Stability, Mechanical Properties and Structural Integrity of MWCNT Filled Bamboo/Kenaf Hybrid Polymer Nanocomposites

**DOI:** 10.3390/ma15020506

**Published:** 2022-01-10

**Authors:** J. M. Prabhudass, K. Palanikumar, Elango Natarajan, Kalaimani Markandan

**Affiliations:** 1 Department of Mechanical Engineering, Sathyabama Institute of Science and Technology, Chennai 600001, India; prabhu.mech@sairamit.edu.in; 2 Department of Mechanical Engineering, Sri Sai Ram Institute of Technology, Chennai 600001, India; 3 Faculty of Engineering, Technology and Built Environment, UCSI University, Kuala Lumpur 56000, Malaysia; kalaimani@ucsiuniversity.edu.my

**Keywords:** bamboo, kenaf, MWCNT, natural fibres, green composite

## Abstract

Recently, there has been an inclination towards natural fibre reinforced polymer composites owing to their merits such as environmental friendliness, light weight and excellent strength. In the present study, six laminates were fabricated consisting of natural fibres such as Kenaf fibre (*Hibiscus cannabinus* L.) and Bamboo fibre, together with multi-walled carbon nanotubes (MWCNTs) as reinforcing fillers in the epoxy matrix. Mechanical testing revealed that hybridization of natural fibres was capable of yielding composites with enhanced tensile properties. Additionally, impact testing showed a maximum improvement of ≈80.6% with the inclusion of MWCNTs as nanofiller in the composites with very high energy absorption characteristics, which were attributed to the high specific energy absorption of carbon nanotubes. The viscoelastic behaviour of hybridised composites reinforced with MWCNTs also showed promising results with a significant improvement in the glass transition temperature (Tg) and 41% improvement in storage modulus. It is worth noting that treatment of the fibres in NaOH solution prior to composite fabrication was effective in improving the interfacial bonding with the epoxy matrix, which, in turn, resulted in improved mechanical properties.

## 1. Introduction

Research on natural fibre reinforced composites has been growing at a relentless pace as it holds the promise of delivering composites with low density (weight) yet high specific properties [1,2,3,4,5]. Some important merits of natural fibres include their abundance in nature, environmental friendliness, low cost and ease of manufacturing. Natural fibres were initially utilised with the intention to yield lighter composites that has the potential to replace glass-fibre reinforced polymer composites, since natural fibres have comparable strength as glass fibre with similar specific modulus [6,7,8,9,10]. Examples of some commonly used natural fibres as reinforcement material in engineering include bast fibres such as hemp, jute, flax, Kenaf, and agava leaf fibres, such as sisal.

Today, as natural fibre reinforced composites are gaining more interest in various applications, research has been diverged towards developing hybrid composites to achieve properties such as ductility, stiffness and strength, which are non-attainable in a single fibre reinforced composite [11]. Besides, the hybridization of natural fibre with another natural or synthetic fibre may merge the advantages of the individual constituent fibre, which results in excellent mechanical properties. For instance, in a study by Ismail et al., the hybrid Kenaf-Bamboo fibre composite showed the best tensile strength and modulus properties compared to single-fibre reinforced composites [12]. Besides, the hybrid composites displayed highest damping factor and sound absorption coefficient; all of which are properties highly desirable for non-load bearing structures where noise control and sound absorption are prime requirements. In another study by Loganathan et al., *Cyrtostachys renda* (CR)-Kenaf fibre-based, hybrid polymer composites were fabricated [13]. The tensile strength of CR and Kenaf composites were 35.2 MPa and 36.7 MPa respectively; and with hybridization of CR and Kenaf the tensile strength was enhanced to 47.8 MPa. Similarly, the tensile modulus of CR and Kenaf composites were 11.94 GPa and 11.59 GPa, respectively; and the hybrid CR-Kenaf composite enhanced the tensile modulus to 14.79 GPa. Similar results were also reported in a study by Hanan et al. using oil palm and Kenaf fibres [14]. In all the aforementioned studies, it is evident that hybridization of fibres as reinforcing fillers in polymer composites may increase the load withstanding capability and, thus, strengthen the composite.

Additionally, the last few decades have witnessed significant research expansion on polymer nanocomposites using various carbonaceous nanofillers such as graphene and carbon nanotubes (CNTs). For instance, CNT as nanofillers in polymer composites have been reported to exhibit high structural and functional properties owing to their high aspect ratio, electrical properties and mechanical strength [15,16]. In a study by Khashaba et al., it was reported that nanofillers have the ability to strengthen synthetic fibre hybrid composites by 30% [17]. Similar findings were reported in other studies [12,18]. Nevertheless, experimental investigation on natural fillers in micro-scale (i.e., Bamboo, Kenaf) and fillers in nanoscale (MWCNTs), and their strengthening mechanism have been least investigated.

In the present study, Kenaf fibre *(Hibiscus cannabinus* L.), a type of bast fibre with high aspect ratio compared to other fibres with superior toughness, strength and stiffness [14] along with Bamboo fibre, which is an extremely light-weight fibre, functionally graded and possesses relatively higher ultimate tensile strength than most plant fibres such as jowar and sisal [15,16] were used as the reinforcing fillers in the Epoxy matrix. The hybrid composites were further reinforced with carbonaceous nanofiller, multi-walled carbon nanotubes (MWCNTs) owing to their high aspect ratio and the excellent structural and functional properties [17,18,19]. To this end, Section 2 of this report details the materials used, fabrication of composites and the various mechanical characterizations conducted. Section 3 highlights the findings obtained from the present study, while Section 4 summarizes the major finding from the present study.

## 2. Materials and Methods

### 2.1. Preparation of Laminates

The current research used Bamboo and Kenaf fibres and multiwall carbon nano-tubes (MWCNT) to reinforce Epoxy resin. In this regard all different combination of fillers were attempted to investigate the effect of single filler reinforcement and hybridization as well. Epoxy HY951 and Aradur 22962 hardener which were used to bind the fibres (in random) were supplied by Herenba Instruments and Engineers, Chennai, India. Untreated Bamboo (*Bambusa vulgaris*) mats and Kenaf (*Hibiscus cannabinus*) mats in 1.2 g/cm^2^ were purchased from a local supplier in Chennai, India. To prevent defects and improve the interfacial adhesion between the fibre and Epoxy, alkali treatment was performed using 5% of NaOH. The Bamboo and Kenaf fibres were then cleaned using distilled water and dried at room temperature. MWCNTs (the reinforcing fillers); NC7000 used in the present study in the form of black powder and spaghetti-like structure at nanoscale with 98% purity, with an average cross-sectional diameter and length of 10 nm and 6–9 µm, respectively, was synthesised using the catalytic chemical vapor deposition method (CCVD) by NANOCYL, Belgium. Laminates having dimensions of 270 mm × 270 mm × 3 mm were prepared at a pressure and temperature of 2500 psi and 230 °C, respectively, using a compression moulding machine (100 tonne capacity). Cooling was undertaken at room temperature. Six laminates of natural fibre reinforced composites were prepared as shown in Table 1.

### 2.2. Characterizations

To evaluate the axial stress withstanding ability of the prepared laminates, the samples were cut according to ASTM D3039 (120 mm × 20 mm × 3 mm) standards. Tensile testing was carried out using a universal testing machine (Instron 4855, Norwood, MA, USA) equipped with a 100 kN capacity load cell. Both the ends of the samples were tabbed to avoid slipping. The respective tensile properties were measured for each laminate. Energy absorbed by each laminate was tested using an impact testing machine (name of equipment, country) according to ASTM D256 standards. To evaluate the transverse loading ability of the prepared laminates, a 3-point bending test was carried out with samples (100 mm × 20 mm × 5 mm) according to ASTM D-790. Dynamic mechanical analysis (DMA) was performed using DMA Q800 (TA Instruments, New Castle, PA, USA) with the 3-point bending clamp setup. The analysis was carried out on a temperature sweep mode from 30 °C to 180 °C at a frequency of 0.1 Hz, preload force of 0.01 N and amplitude of 15 µm. Unless otherwise stated, 3 samples were fabricated and tested for each laminate condition to ensure reproducibility of test results.

Thermo gravimetric analysis (TGA) was carried out using Netzsch STA 449F3 instrument (Netzsch STA 449F3, Selb, Germany) to evaluate the expansion and contraction of the laminates due to fibre swelling from moisture absorption. It was undertaken in an alumina crucible that was heated in flowing air (260.3 mL/min) and nitrogen (250.0 mL/min) from 35 °C to 1000 °C at a heating rate of 10 °C/min. The vibrational spectra of the prepared composites were recorded using Fourier transform infrared (FTIR) spectrometer (FT/IR-6300typeA FTIR spectrometer, JASCO Co., Tokyo, Japan) while the viscous behaviour of the laminates having dimensions of 10 mm × 10 mm × 4 mm was measured using the cantilever method from 25 °C to 168 °C at a heating rate of 100 °C/min and maximum frequency of 5Hz. Scanning electron microscopy (SEM) was performed using JSM-7600F (JEOL Inc., Tokyo, Japan) with an accelerating voltage of 2–5 kV on the surfaces of the fractured surfaces. The samples were first coated with ~4 nm of Pt using Hitachi S-3000N machine and examined to understand the microstructure of the broken samples. Raman spectroscopy analysis was performed using stellar-Pro ML150 laser coupled to an optical microscope (Leica DM 2500 M) with Renishaw He-Ne laser with 633 nm wavelength as excitation source.

## 3. Results and Discussions

### 3.1. Thermal Studies (TGA and DTG Analysis)

**Figure 1:**
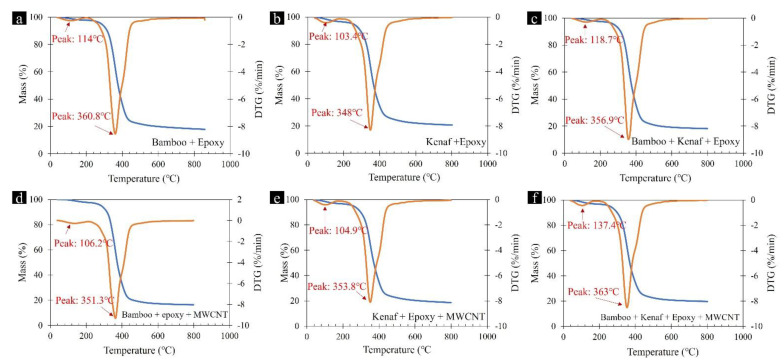
TGA and DTA curves of (**a**) Bamboo + Epoxy (**b**) Kenaf + Epoxy (**c**) Bamboo + Kenaf + Epoxy (**d**) Bamboo + Epoxy + MWCNT (**e**) Kenaf + Epoxy + MWCNT (**f**) Bamboo + Kenaf + Epoxy + MWCNT composites between 0 °C and 900 °C.

The combined TGA and DTG data from 35 °C to 800 °C of the six prepared laminates are shown in Figure 1. The first-derivative curve of the composites lies between the onset temperature of 311 °C to an end set temperature of 550 °C. The observed results from the thermo-oxidative decomposition of the laminates are presented in Table 2, where it is seen that the laminates exhibited two stages of mass losses ranging from 36.6 °C to 200 °C and from 200 °C to 800 °C. The residual mass of the compounds ranged from 18.25 to 20.76%. Similar two-stage mass loss and residual mass percentages have been reported in other Bamboo based composite studies [19,20,21]. Comparatively smaller weight loss and DTG peak in the first stage can be related to water loss from the laminates, whereas the major weight loss that occurred during the second stage is attributed to the thermal degradation of the lignocellulosic components of the fibre (cellulose decomposition). The residual mass could be from the char or other end-products from the decomposition reaction. Besides, it is also seen that the mass loss of laminates consisting of Kenaf (b–f) was higher than Bamboo-based laminates (a, c, e and f), which can be attributed to the higher moisture content in Kenaf fibres. Bamboo based laminates also displayed better thermal stability (i.e., higher decomposition temperature) in comparison to Kenaf based laminates. Although the laminates reinforced with MWCNT (d–f) showed similar residual mass as other laminates, it is worth noting that some MWCNT-reinforced laminates exhibited higher decomposition temperature (maximum of 363 °C), which indicates that high thermal stability can be attained with the inclusion of MWCNT.

### 3.2. FTIR Characterization

**Figure 2:**
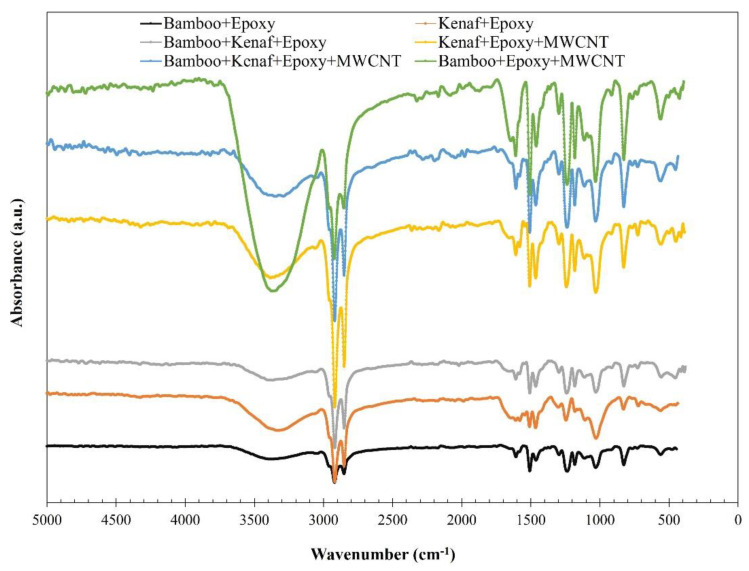
FT-IR spectra of Epoxy laminates consisting of natural fibres and/or MWCNT fillers.

The vibrational frequencies from FT-IR analysis and the resultant peak values are shown in Figure 2 and Table 3, respectively. The vibrational broad band between 3600 and 3200 cm^−1^ region represents the O–H stretching vibrations of the cellulose fibres such as Bamboo and Kenaf [22]. The composites exposed significant peaks with respect to the following modes of vibrations such as C-H stretching (2924 cm^−1^), C=O stretching (1641 cm^−1^), aromatic ring (1591, 1505, 1467 cm^−1^), C-H deformations, C-O stretch (1242 cm^−1^), C-O stretching of lignin, ester groups (1180 cm^−1^), C-O deformations (1018 cm^−1^) and p-hydroxyphenyl C-H out of plane (827, 822, 698 cm^−1^). Similar findings have been reported in other studies [23,24]. MWCNTs have shown the side wall C=C bond peat at 1641 cm^−1^ [25]^,^ which ascertains the interaction (adhesion) of MWCNT within the composites. All the spectra of these composites have confirmed the compositions and interaction between the Bamboo, Kenaf and natural cellulose functional groups with the Epoxy resin. Besides, the polymerization of Epoxy resin and product oxidation were confirmed by the absence of Epoxy resin’s significant peak at 915 cm^−1^. A detailed FT-IR spectrum of all laminates labelled with their respective vibrational bands are shown in the Supplementary Materials.

### 3.3. Effect of MWCNT Addition: Raman Analysis

**Figure 3:**
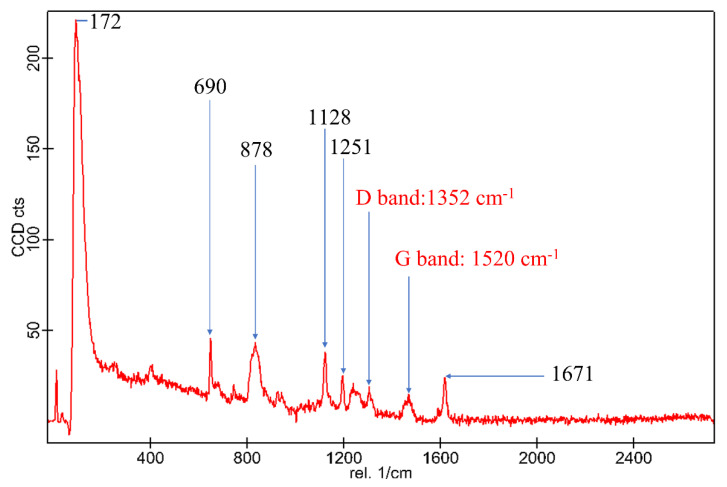
Raman spectrum of CNT reinforced Epoxy composites.

The structural changes on the molecular level of MWCNT based Epoxy composites were investigated using Raman spectroscopy. From Figure 3, the Raman bands located at 878 cm^−^^1^ and 1128 cm^−^^1^ were assigned to the resin backbone vibrations, whereas Raman bands corresponding to Epoxy ring deformations and Epoxy ring breathing were located at 690 cm^−^^1^ and 1251 cm^−^^1^ respectively [26,27]. The Raman band at 1671 cm^−^^1^ can be assigned to the amide I vibration, predominant of C=O stretching of the amide group [28]. The band at 172 cm^−^^1^ can be associated with the highly crystalline cellulose structure (i.e., hydrogen bonds between cellulose molecules) originating from Bamboo and Kenaf fibres. Similar band have been reported for Avicel (SA21) Valonia and tunicate (SA18) celluloses [29]. Raman spectrum of graphene platelets show two strong peaks at 1352 cm^−^^1^ and 1530 cm^−^^1^, which correspond to the D and G-bands, respectively, and are consistent with typical characteristics of graphitic MWCNTs [30]. The band G-band of MWCNTs in the present study indicate the presence of crystalline graphite, sp^2^, which shows that the nanotubes are highly graphitised. The intensity of the D-band to G-band (I_D_/I_G_) semi-quantitatively indicate the structural integrity of MWCNTs in the Epoxy matrix. In the present study, the I_D_/I_G_ ratio was 1.12, which ascertains that the MWCNTs were not imposed to significant amount of defects [31].

### 3.4. Mechanical Characterization

#### 3.4.1. Tensile Behaviour and Fracture Mechanism

**Figure 4:**
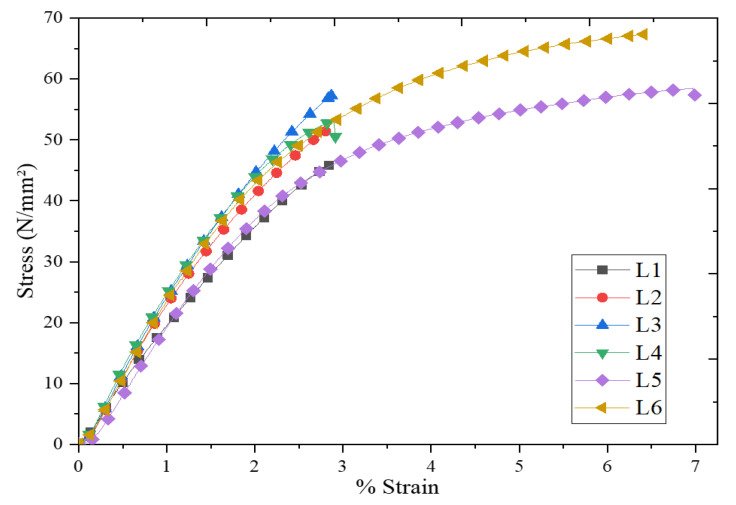
Stress-strain curves of laminates fabricated in the present study (L1: Bamboo + Epoxy; L2: Kenaf + Epoxy; L3: Bamboo + Kenaf + Epoxy; L4: Bamboo + Epoxy + MWCNT; L5: Kenaf + Epoxy + MWCNT; L6: Bamboo + Kenaf + Epoxy +MWCNT).

The stress-strain curves of the laminates are shown in Figure 4 where it can be seen that initially tensile stresses varied linearly with deformation or increased strain. The proportionality continued until the maximum value, where the linearity eases out at the stress value. Further increase in load ceases the proportionality to reach the ultimate disproportionately, after which the catastrophic fracture occurred. Maximum tensile strength was observed in laminate 6 (Kenaf + Bamboo + MWCNT + Epoxy) with a value of 67.349 N/mm^2^ whereas lowest tensile strength was observed in laminate 1 (Bamboo + Epoxy) with a value of 45.85 N/mm^2^. Similar high-tensile strength value of Kenaf + Bamboo fibre + MWCNT reinforced Epoxy composites were also reported in other studies [12,13,32]. In a study by Alavudeen et al., it was reported that Kenaf fibres are stronger and stiffer in the longitudinal direction which could lead to the greater stress uptake and higher mechanical strength of the composites [33]. Besides, it should be noted that hybridization of high elongation fibres with low elongation fibres in Epoxy matrix can improve the required strain level for propagation of fibre breakage, since the higher elongation fibre will act as crack arrestor at micromechanical level.

**Figure 5:**
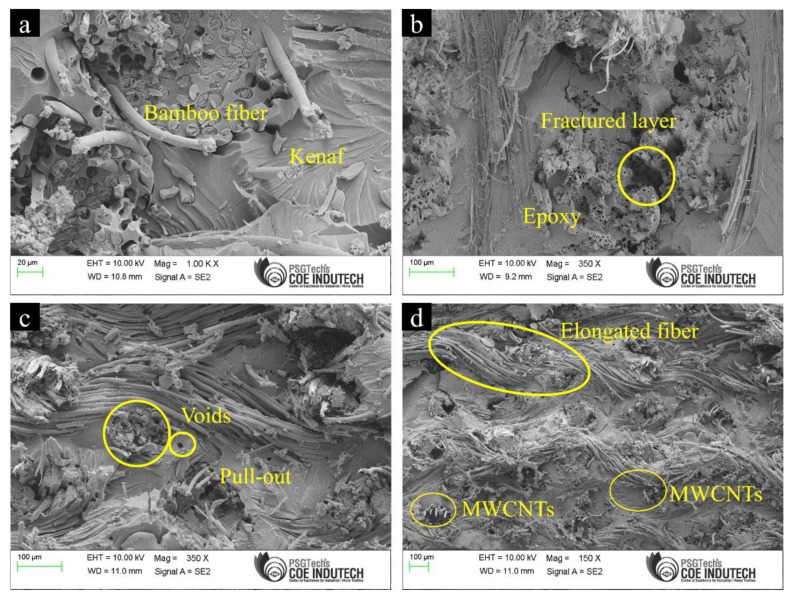
SEM images of the fracture surfaces from tensile testing of laminate 6 (Bamboo + Kenaf + Epoxy + MWCNT), showing (**a**) the reinforcement of Bamboo and Kenaf fibre in the Epoxy matrix; (**b**) the fractured layer from tensile testing; (**c**) fibre pull-out mechanism in the composite, and; (**d**) reinforcement with MWCNT and elongated fibre in the Epoxy matrix.

Figure 5 shows the SEM images of the fracture surfaces from tensile testing of laminate 6 (Bamboo + Kenaf + Epoxy + MWCNT). From Figure 5a, it can be seen that the fibres are disorderly distributed within the matrix; where their disorderliness led to pull-outs, detachment and debonding. Large smooth zones are also visible along the isolated broken fibres. It should also be noted that Kenaf reinforced composites showed comparatively better tensile properties in comparison to Bamboo reinforced composites. This is because compressing the entire stack at the time of laying up the composites leads to long punched fibres and Kenaf fibres have the ability to retain the length during the compression process which differs from Bamboo fibres. These long fibres are capable of carrying more load due to the increase in transfer length and an appreciable adhesion with the matrix, which results in enhanced mechanical properties of the composites. In fact, Kenaf as a single reinforcement in the Epoxy matrix was capable of producing a high tensile strength of 51.4 N/mm^2^, even without the addition of MWCNT fillers. Table 4 summarizes the tensile properties of composites in the present study in comparison to other studies.

The addition of MWCNTs elevated the strength of the composite by 3.7%, which can be attributed to the efficiency of load transfer and the interfacial bonding between MWCNT, other fillers and the Epoxy matrix. The high surface area per unit volume of MWCNT led to a good interfacial bond. which is essential to ensure the effective stress transfer from the Epoxy matrix to the fibres, through which the fibre’s strength can be utilised to its full potential in the composite [34,35]. The treatment of the fibres in NaOH solution prior to composite fabrication has been proven to improve the interfacial bonding with the Epoxy matrix. which results in enhanced tensile properties. This is because, NaOH effectively removed the lignin present in the fibres, which, in turn, splits the fibres into shorter ones that reduce the voids present, thus, providing better bonding between the fibre and Epoxy matrix [3]. Nevertheless, duration of fibre treatment in NaOH solution is an important factor since prolonged treatment may weaken and degrade the property of the fibres.

#### 3.4.2. Impact Behaviour and Fracture Mechanism

**Figure 6:**
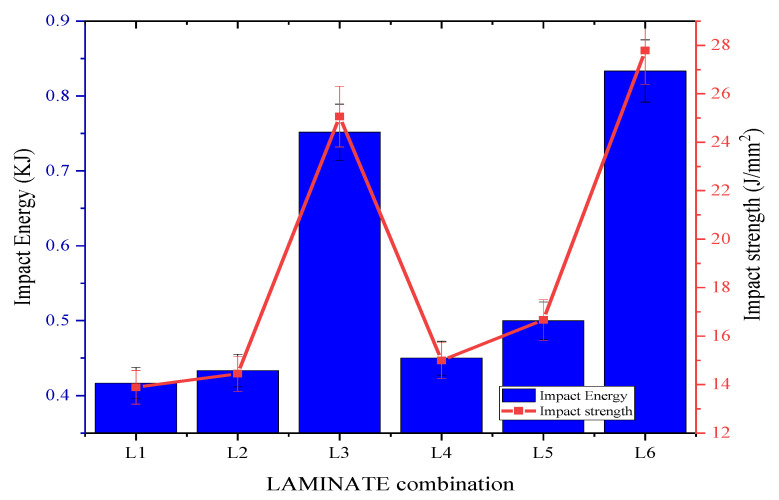
Energy absorbed and impact strength of laminates subjected to Izod’s impact test.

Izod’s impact test was conducted on the composite samples to ascertain their capability of withstanding sudden load. From Figure 6, it can be seen that the impact strength of laminate 1 (Bamboo + Epoxy) and laminate 2 (Bamboo + Kenaf) were 13.9 J/mm^2^ and 14.4 J/mm^2^ respectively. The slightly higher impact strength of Kenaf reinforced composite can be attributed to the woven Kenaf structure compared to the non-woven Bamboo; where woven structure fibres render better impact strength than the random fibres orientation [36]. Upon hybridization (i.e., laminate 3: Bamboo + Kenaf + epoxy); the impact strength increased by 80.6% and 74.3% in comparison to laminate 1 and 2 respectively. The significant improvement in the hybridised composite can be attributed to the incorporation of both Kenaf and Bamboo in Epoxy, which induced higher energy absorption capability due to the excellent interfacial bonding. The excellent interfacial bonding of the Kenaf and Bamboo fibre with the Epoxy matrix hinders the initial break, the subsequent break pinning mechanism and the extension of crack within the composite when force is applied [4]. In comparison, laminate 6 (Kenaf + Bamboo + MWCNT + Epoxy) showed the best resilience to sudden shock with excellent energy absorption characteristics in comparison to other composites, where there were ≈99.8% and ≈100% improvement in impact energy absorption and strength, respectively, compared to the values reported by laminate 1 and laminate 2 in average. The significant improvement in impact properties upon addition of MWCNTs is due to excellent impact energy absorption capability of MWCNTs compared to the Epoxy matrix; the more energy absorbed by the MWCNTs reinforced laminate indicates a tougher composite material.

In a study by Shirvanimoghaddam et al., it was reported that for polymer blends, factors such as interparticle distance or matrix ligament thickness determines the brittle to tough transition of the polymer blend [37]. As such the addition of fillers in the present study could have reduced the interparticle distance, which, in turn, toughened the Epoxy matrix. It was also reported that smaller particle size will be highly beneficial than the larger particles in toughening the polymer matrix by predominant toughening mechanisms such as stress concentration or shear yielding. In the present study, MWCNTs as nanofiller played an essential role in dissipating energy through fracture at the same time applied stress on the matrix to increase the strength of the matrix in a synergistic manner. It has been reported that the reinforcing phase in composites with interpenetrating phase structures allows additional plastic deformation, which results in increased strength and toughness [38]. It was also suggested in the study by Shirvanimoghaddam et al. [37] that altering the crystallinity of the polymer matrix can enhance the toughening efficiency. For instance, altering the crystalline state form of polypropylene from α to β can improve the toughening efficiency of the polymer where β- crystal structure creates a bundle-like structure to enhance the energy dissipation, thereby toughening the composite [39]. In the present study, it is assumed that the addition of MWCNTs to the Epoxy matrix migrated the MWCNTs to the Epoxy interface, which thereby formed a network structure to increase the fracture toughness, as observed from the increase in the impact strength values. Similar findings were reported in a study by Bai et al. [40]. Crystallinity, phase changes and nucleation of Epoxy matrix with the addition of micro- and nano-scale fillers to toughen the composite are left to future studies.

**Figure 7:**
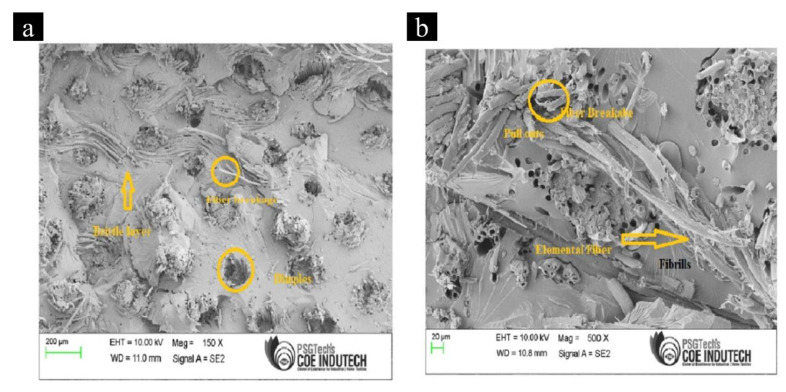
SEM images of (**a**) laminate 1: Bamboo + Epoxy and (**b**) laminate 6: Kenaf + Bamboo + MWCNT + Epoxy.

Figure 7 shows the fracture surfaces of the sample after Izod’s impact test, wherein Figure 7a shows the SEM images of Laminate 1 (Bamboo + Epoxy), while Figure 7b shows Laminate 6 (Bamboo + Kenaf + Epoxy + MWCNT). In both Figure 7a,b, it was observed that Bamboo fibres, Kenaf fibres and MWCNTs had good interfacial bonding with the Epoxy matrix. Fibre breakages were more prominent in Bamboo reinforced composites as shown in Figure 7a, a phenomenon rarely observed in Kenaf fibre reinforced composite. The high impact strength and energy absorption characteristics of Kenaf fibre reinforced composites (L3 and L6) can be attributed to the excellent adhesion of the long fibres with the Epoxy matrix, which increases the energy absorption capability of the composite. Besides, the Bamboo fibres are prone to breaking under compressive loads during the lay-up process, which is not the case with Kenaf fibres. The broken Bamboo fibres along with the additional gap created by the broken fibres generate a void in between the fibres and within the Epoxy matrix which acts as the weak “leak link” thereby reducing the energy absorption ability of the composite. Moreover, the addition of MWCNTs as fillers further enhanced the energy absorption ability and impact strength of the composites. Other than the load transfer efficiency by MWCNTs, the high energy absorption of the composite can be attributed to the high specific energy absorption of MWCNT itself, i.e., 1.62 GJ m^−3^ [41].

#### 3.4.3. Flexural Behaviour and Fracture Mechanism

**Figure 8:**
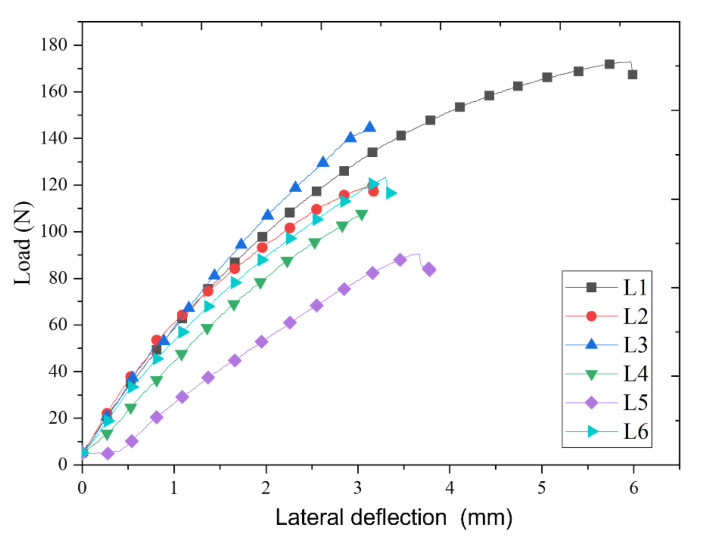
Flexural strength of laminates subjected to three point bending test.

Flexural strength of a material is referred to as the maximum bending stress required to break a beam under a three-point bending load. The flexural behaviour of composites fabricated in the present study is shown in Figure 8; where maximum transverse load of 172.95 N was observed with 5.9 mm deflection on laminate 1 (Bamboo + Epoxy) composite, while a minimum value of 90.546 N was obtained with 3.78 mm deflection in laminate 5 (Kenaf + Epoxy + MWCNT) composite. Besides, laminate 3 (Bamboo + Kenaf + Epoxy) reported a transverse load of 144.58 N with minimum deflection of 3.04 mm, a specific property that demonstrates the importance of fibre hybridization in improving the transverse load-carrying capacity and reduced deflections of the composites. Conversely, the addition of MWCNT did not show any significant improvement in the flexural strength of the composites. This can be related to the possible agglomeration of MWCNTs inside the Epoxy matrix where even a small agglomeration of MWCNT can behave as the failure concentration point which hampers the flexural properties of the entire composite network. The agglomeration of MWCNTs is possible owing to the large surface area due to its nano-scale diameter with a high aspect ratio of >1000 [16,42].

**Figure 9:**
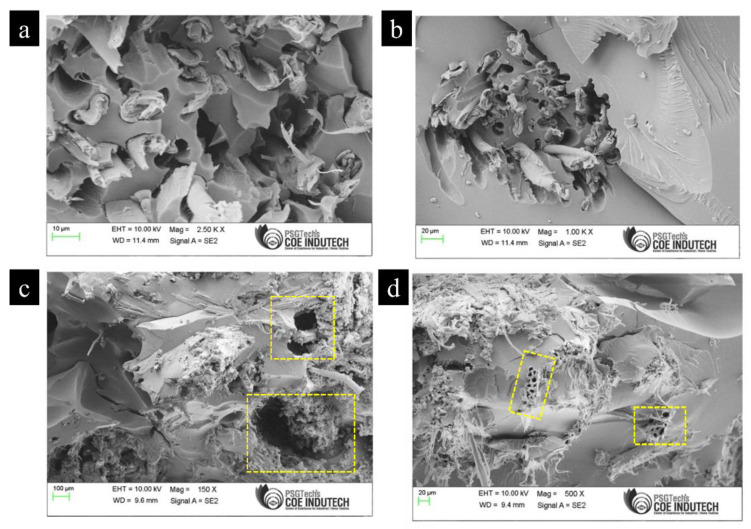
SEM images of samples subjected to flexural testing (**a,b**) laminate 3 (Bamboo + Kenaf + Epoxy) (**c,d**) laminate 6 (Kenaf + Bamboo + Epoxy + MWCNT).

Figure 9 shows the SEM images of the of samples subjected to flexural testing where Figure 9a,b show laminate 3 (Bamboo + Kenaf + Epoxy) whereas Figure 9c,d show laminate 6 (Kenaf *+* Bamboo + Epoxy + MWCNT). Phenomena such as fibre breakage, pull-outs, voids and matrix cracking were all clearly observed from these figures. From Figure 9a,b fibre breakages were visible which indicates the adequate interfacial bonding between fibre and the matrix. In a study by Ismail et al., it was reported that upon flexural testing, fibres in the horizontal direction showed breakage whereas fibre in the vertical direction exhibited pull-out [36]. However, such phenomenon was not observed in the present study where a combination of fibre breakage and pull-out were both observed regardless of the fibre’s orientation direction. This factor could possibly explain the lower flexural properties of laminate 3 in comparison to laminate 1 (Bamboo + Epoxy). On the other hand, Figure 9c,d show the MWCNTs reinforced composites where sites of MWCNTs agglomeration, which behaves as the failure concentration point were visible along with the presence of broken fibres with deep pits in a broad area, all of which hampered the flexural properties of the composite. Based on all the results presented above, the mechanical properties of the laminates are summarised as shown in Table 5. It is evident that while tensile and impact strength of the hybrid polymer composites were improved with the addition of MWCNTs; flexural strength was significantly reduced for all the aforementioned reasons.

#### 3.4.4. Dynamic Mechanical Analysis (DMA)

**Figure 10:**
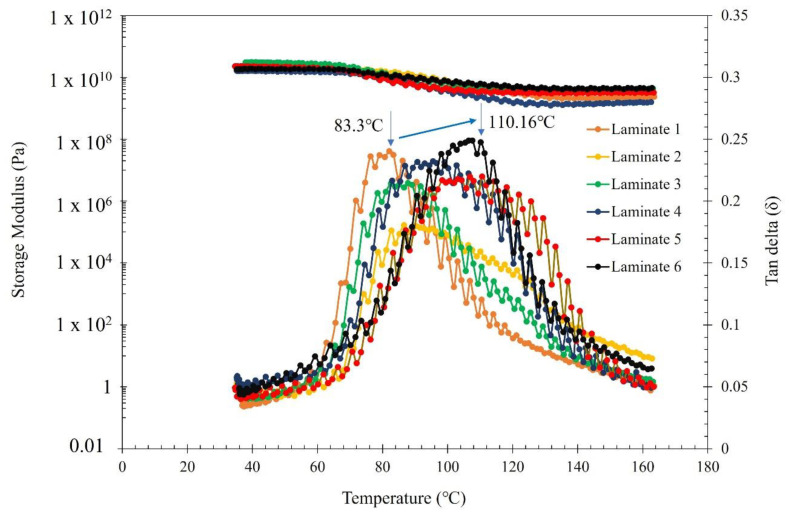
Storage modulus and tan δ as a function of temperature for laminates fabricated in the present study.

Figure 10 shows the storage modulus (Eʹ) and tan δ as a function of temperature for all the six laminates fabricated in the present study. It can be seen that laminate 6 (Kenaf + Bamboo + MWCNT + Epoxy) exhibited the highest storage modulus values across the temperature range of 30 °C to 180 °C in comparison to other laminates. For instance, there was ≈40.6% improvement in Eʹ in laminate 6 in comparison to laminate 1 (Bamboo + Epoxy) which ascertains the excellent interfacial adhesion of MWCNTs with the Epoxy matrix via intermolecular forces. Besides, the addition of natural fibres such as Kenaf that has high stiffness (≈40 GPa) [43] could have contributed to the overall stiffness of the composite. Similar findings have been reported in another study using natural fibre such as bagasse, rice straw, rice husk and pine [44]. As such, the application of sinusoidal dynamic force to the highly stiff Bamboo + Kenaf + Epoxy + MWCNT has shown to exhibit remarkable stress-transfer effects.

Glass transition temperature (Tg) of the laminates were investigated using DMA which was determined by the maximum peak of tan δ. It is clearly evident that the Tg of laminates increased from 83.3 °C to a maximum value of 110.2 °C when the Epoxy matrix was reinforced with Kenaf, bamboo and MWCNTs. Besides, it is also observed that the Tg of laminates 4, 5 and 6 (i.e., laminates with MWCNTs) were higher than laminate 1, 2 and 3 (i.e., without MWCNTs reinforcement). This is a clear indication that the incorporation of MWCNTs have played a significant role in increasing the Tg of the composites. The increase in Tg after incorporation of MWCNTs is attributed to the reinforcement effect, which tightens the whole network and hinders mobility of polymer chains, an indication of good adhesion between the MWCNTs, bamboo fibre, Kenaf fibre and the Epoxy matrix. Similar findings have been reported in other studies with the incorporation of MWCNTs as reinforcement filler [45,46].

## 4. Conclusions

In conclusion, hybridization of natural fibre with reinforcing effect from MWCNTs in Epoxy composites showed improved mechanical properties. For instance, maximum tensile strength of 67.3 MPa was achieved in Kenaf + Bamboo + Epoxy + MWCNT composite. Hybridization of high elongation fibres with low elongation fibres in the Epoxy matrix improved the strain level required for propagation of fibre breakage where higher elongation fibre was capable of arresting the crack at micromechanical level. Besides, Kenaf + Bamboo + Epoxy + MWCNT hybrid composites showed the best resilience to sudden shock with excellent energy absorption characteristics in comparison to other composites attributed to the excellent load transfer efficiency of MWCNT. Furthermore, incorporation of MWCNTs played an essential role in increasing Tg of the composites by providing the tightening effect to the whole network, which, in turn, hinders mobility of the polymer chains. The insights derived from our study indicate that the hybridization of natural fibres in Epoxy matrix, together with the addition of MWCNTs as nanofiller reinforcement has the potential to be a leading material for applications requiring high strength and excellent impact energy absorption.

## Figures and Tables

**Table 1:** Composition of prepared laminates with varying amounts of natural fibres, Epoxy resin and MWCNT fillers. Superscripts ^b^ and ^k^ indicate compositions of Bamboo and Kenaf fibre, respectively.

Laminate	Composition	Fibre (g)	Resin (g)	Filler (g)	Total (g)
Laminate 1	Bamboo + Epoxy	105	195	0	300
Laminate 2	Kenaf + Epoxy	105	195	0	300
Laminate 3	Bamboo + Kenaf + Epoxy	45 ^b^ & 60 ^k^	195	0	300
Laminate 4	Bamboo + Epoxy + MWCNT	105	189	6	300
Laminate 5	Kenaf + Epoxy + MWCNT	105	189	6	300
Laminate 6	Bamboo + Kenaf + Epoxy + MWCNT	45 ^b^ & 60 ^k^	189	6	300

**Table 2:** TGA/DTG data summary of Epoxy laminates consisting of natural fibres and/or MWCNT fillers.

	Composite	Mass Change (%)		DTG Peak Values (°C)		Residual Mass (%)	Onset Temp (°C)
		36.6 °C to 200 °C	200 °C to 800 °C	36.6 °C to 200 °C	200 °C to 800 °C		
a	Bamboo + Epoxy	2.14	79.62	113.9	360.8	18.25	319.9
b	Kenaf + Epoxy	3.44	75.80	103.4	348.0	20.76	311.2
c	Bamboo + Kenaf + Epoxy	2.61	79.24	118.7	356.9	18.14	319.8
d	Kenaf + Epoxy + MWCNT	3.13	78.15	106.2	351.3	18.69	312.1
e	Bamboo + Kenaf + Epoxy + MWCNT	3.07	77.17	104.9	353.8	19.77	315.8
f	Bamboo + Epoxy + MWCNT	2.32	81.26	137.4	363.0	16.43	322.8

**Table 3:** FTIR vibrational spectra of prepared composites.

Composite	FTIR Frequencies (cm^−1^)	Observation
(a) Laminate 1: Bamboo + Epoxy	3363, 2924, 2857, 1591, 1505, 1467, 1242, 1180, 1018, 827, 822, 698.	Epoxy ring peak—915 cm^−1^ vanished
(b) Laminate 2: Kenaf + Epoxy	3363, 2924, 2857, 1641 (broad), 1591, 1505, 1467, 1242, 1180, 1018, 827, 822, 698.	Epoxy ring peak—915 cm^−1^ vanished
(c) Laminate 3: Bamboo + Kenaf + Epoxy	3363, 2924, 2857, 1641 (broad), 1591, 1505, 1467, 1242, 1180, 1018, 827, 822, 698.	Epoxy ring peak—915 cm^−1^ vanished
(d) Laminate 4: Bamboo+Epoxy + MWCNT	3363, 2924, 2857, 1641 (broad), 1591, 1505, 1467, 1242, 1180, 1018, 827, 822, 698.	Epoxy ring peak—915 cm^−1^ vanished
(e) Laminate 5: Kenaf + Epoxy + MWCNT	3363, 2924, 2857, 1641 (broad), 1591, 1505, 1467, 1242, 1180, 1018, 827, 822, 698.	Epoxy ring peak—915 cm^−1^ vanished
(f) Laminate 6: Bamboo + Kenaf + Epoxy + MWCNT	3363, 2924, 2857, 1641 (broad), 1591, 1505, 1467, 1242, 1180, 1018, 827, 822, 698.	Epoxy ring peak—915 cm^−1^ vanished

**Table 4:** Summary of tensile properties reported in other studies and the present study.

Polymer	Microfiller		Nanofiller		Tensile Strength (MPa)	Tensile Modulus (GPa)	Reference
	Fillers	Filler Concentration (wt.%)	Filler	Filler Concentration (wt.%)			
Epoxy	Bamboo + Kenaf	40	Nanoclay	1	55.8	2.51	[4]
Polyester	Sisal + Jute	NA	Glass fibre	NA	200	NA	[5]
Phenolic resin	Cyrtostachys renda + Kenaf	50	CNTs	0.5	47.96	14.79	[13]
Epoxy	Bamboo + Kenaf	35	MWCNTs	2	67.3	1.05	Present study

**Table 5:** Summary of mechanical properties of composites fabricated in the present study.

	Laminates	Tensile Test		Impact Test		Flexural Test	
		Stress (N/mm^2^)	Strain (%)	Impact Energy (kJ)	Impact Strength (J/mm^2^)	Maximum Load (N)	Deflection (mm)
L1	Bamboo + Epoxy	45.852	2.84	0.417	13.889	172.95	5.99
L2	Kenaf + Epoxy	51.403	2.8	0.433	14.444	119.584	3.22
L3	Bamboo + Kenaf + Epoxy	57.725	2.867	0.752	25.056	144.599	3.04
L4	Bamboo + Epoxy+ MWCNT	53.253	2.913	0.450	15.000	144.599	5.89
L5	Kenaf + Epoxy + MWCNT	58.491	6.993	0.500	16.667	90.546	3.78
L6	Bamboo + Kenaf + Epoxy + MWCNT	67.349	6.413	0.833	27.778	123.41	3.35

## Data Availability

Dataset related these studies, findings and results as reported are included in the manuscript itself. It is also available from the corresponding author on reasonable request.

## Supplementary Materials

The following supporting information can be downloaded at: https://www.mdpi.com/article/10.3390/ma15020506/s1. Figure S1: FTIR Analysis of Laminate 1: Bamboo + Epoxy; Figure S2: FTIR Analysis of Laminate 2: Kenaf + Epoxy; Figure S3: FTIR Analysis of Laminate 3: Bamboo + Kenaf + Epoxy; Figure S4: FTIR Analysis of Laminate 4: Bamboo + Epoxy + MWCNT; Figure S5: FTIR Analysis of Laminate 5: Kenaf + Epoxy + MWCNT; Figure S6: FTIR Analysis of Laminate 6: Bamboo + Kenaf + Epoxy + MWCNT.
