# Supplementary material for: Enhanced Thermal Stability, Mechanical Properties and Structural Integrity of MWCNT Filled Bamboo/Kenaf Hybrid Polymer Nanocomposites
Source: Materials (Basel). 2022 Jan 10;15(2):506. doi: 10.3390/ma15020506 (PMC8777606; doi:10.3390/ma15020506)
Supplement: Supplementary file 1 [file materials-15-00506-s001.zip › materials-1501795-supplementary.pdf]

Supplementary File

Enhanced Thermal Stability, Mechanical Properties and Structural Integrity of MWCNT Filled Bamboo/Kenaf Hybrid Polymer Nanocomposites

J.M.Prabhudass [1,2], K.Palanikumar [2,*], Elango Natarajan [3,*], Kalaimani Markandan [3]

1. Department of Mechanical Engineering, Sathyabama Institute of Science and Technology, 600001 Chennai, India; prabhu.mech@sairamit.edu.in
2. Department of Mechanical Engineering, Sri Sai Ram Institute of Technology, 600001 Chennai, Tamil Nadu, India
3. Faculty of Engineering, Technology and Built Environment, UCSI University, 56000, Kuala Lumpur, Malaysia; kalaimani@ucsiuniversity.edu.my

* Correspondence: palanikumar@sairamit.edu.in (K.P.); elango@ucsiuniversity.edu.my (E.N.)

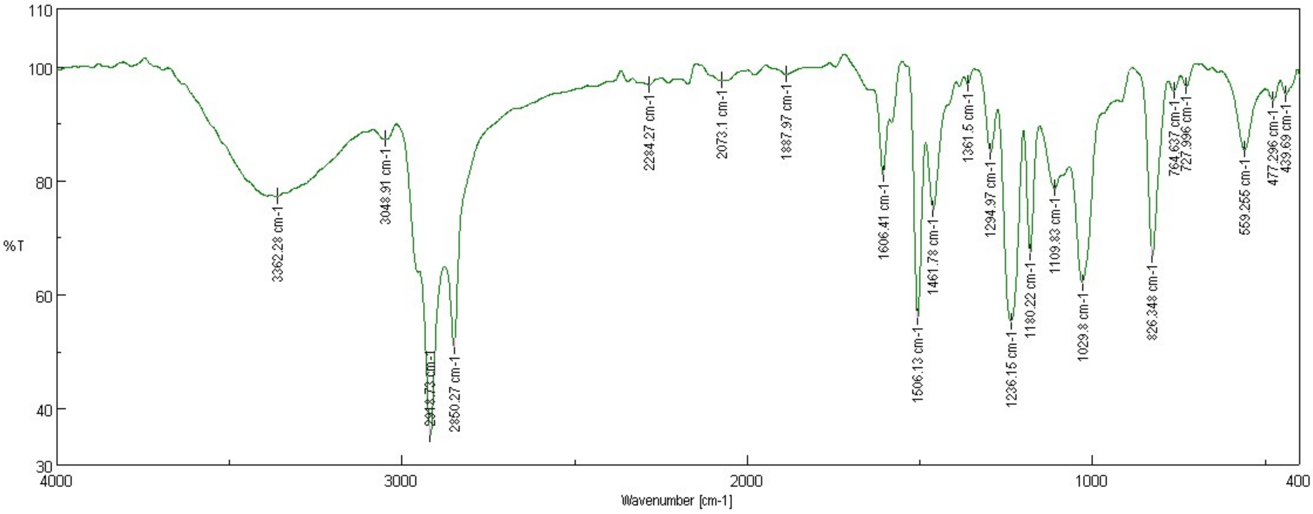

Figure S1: FTIR Analysis of Laminate 1: Bamboo + Epoxy

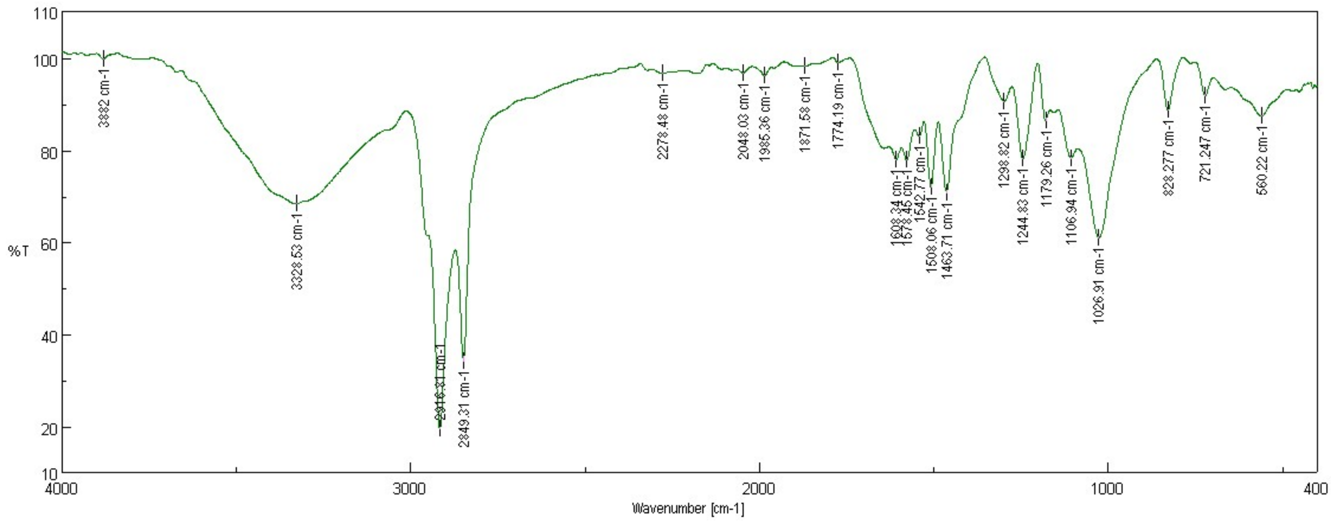

Figure S2: FTIR Analysis of Laminate 2: Kenaf + Epoxy

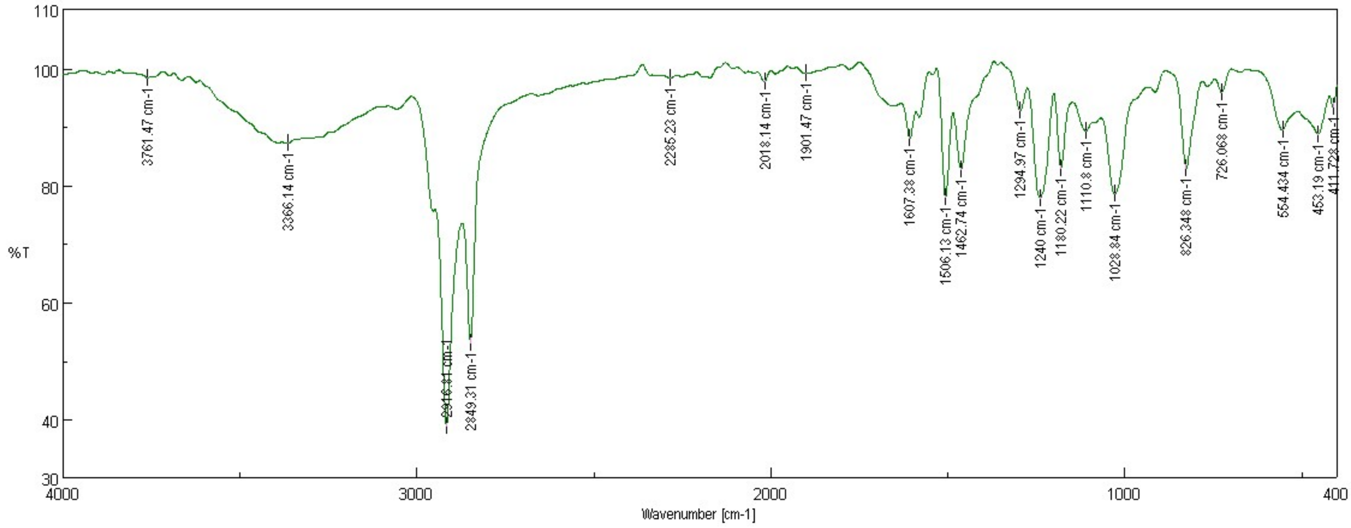

Figure S3: FTIR Analysis of Laminate 3: Bamboo + Kenaf + Epoxy

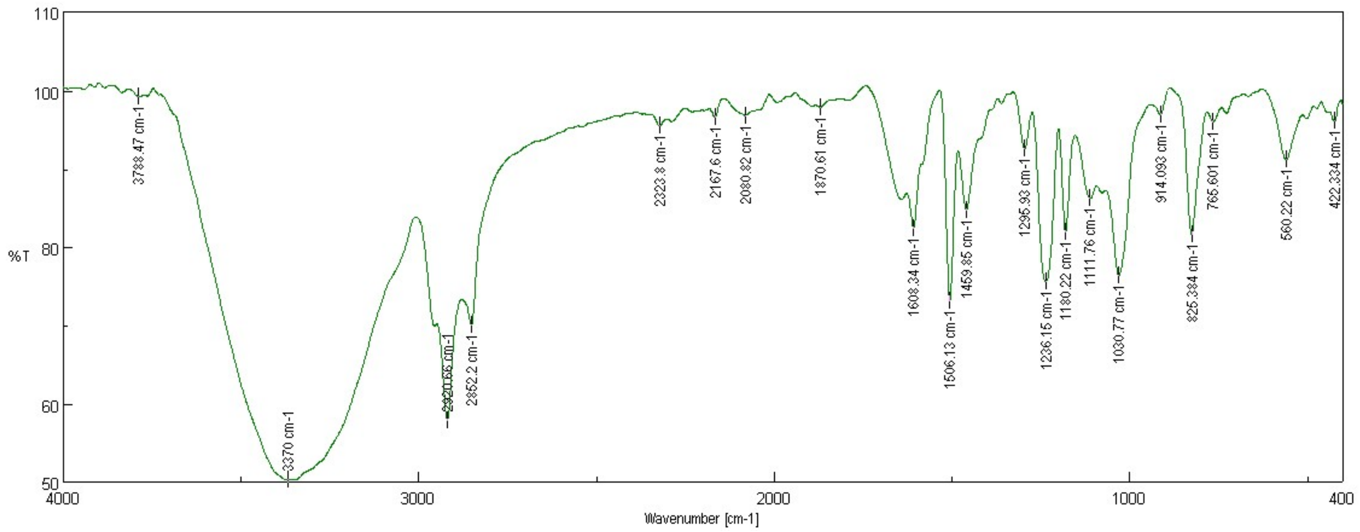

Figure S4: FTIR Analysis of Laminate 4: Bamboo + Epoxy + MWCNT

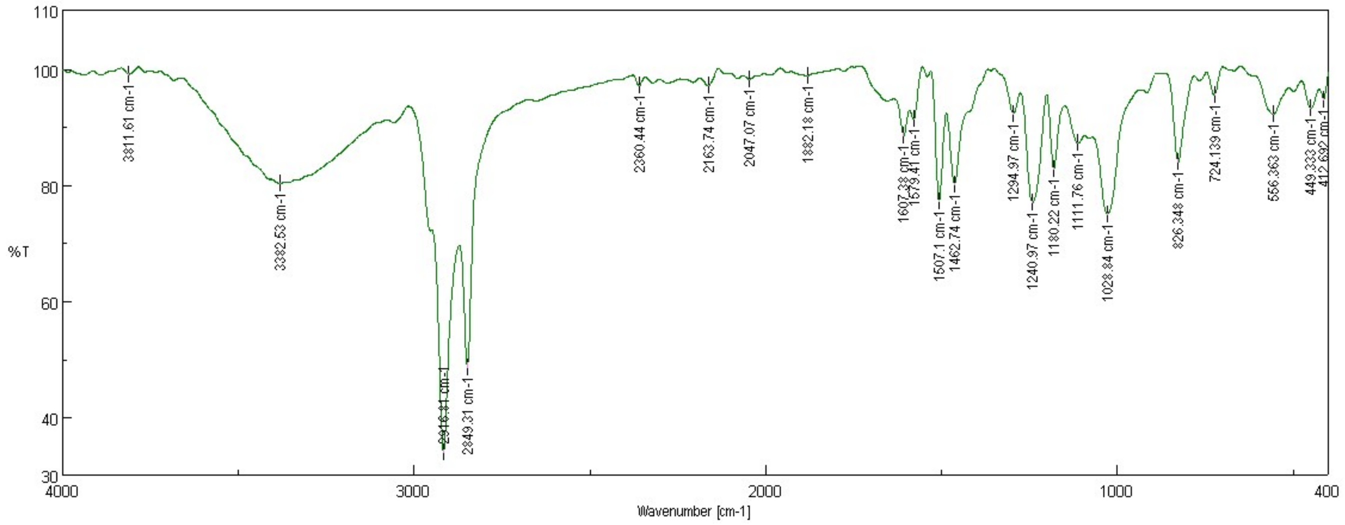

Figure S5: FTIR Analysis of Laminate 5: Kenaf + Epoxy + MWCNT

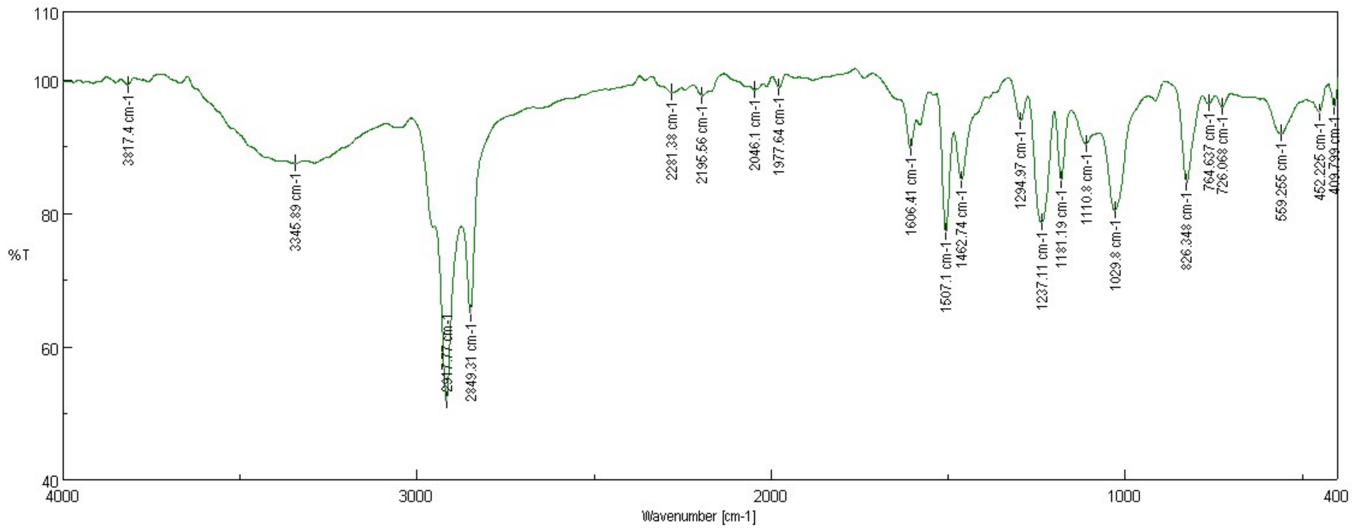

Figure S6: FTIR Analysis of Laminate 6: Bamboo + Kenaf + Epoxy + MWCNT
